# Inverse dose-rate effect of ionising radiation on residual 53BP1 foci in the eye lens

**DOI:** 10.1038/s41598-019-46893-3

**Published:** 2019-07-18

**Authors:** Stephen G. R. Barnard, Roisin McCarron, Jayne Moquet, Roy Quinlan, Elizabeth Ainsbury

**Affiliations:** 1 Public Health England, Centre for Radiation, Chemical and Environmental Hazards, Chilton, Didcot, Oxon UK; 2 Durham University, Department of Biosciences, Durham, UK

**Keywords:** Mechanisms of disease, Animal disease models, Occupational health

## Abstract

The influence of dose rate on radiation cataractogenesis has yet to be extensively studied. One recent epidemiological investigation suggested that protracted radiation exposure increases radiation-induced cataract risk: cumulative doses of radiation mostly <100 mGy received by US radiologic technologists over 5 years were associated with an increased excess hazard ratio for cataract development. However, there are few mechanistic studies to support and explain such observations. Low-dose radiation-induced DNA damage in the epithelial cells of the eye lens (LECs) has been proposed as a possible contributor to cataract formation and thus visual impairment. Here, 53BP1 foci was used as a marker of DNA damage. Unexpectedly, the number of 53BP1 foci that persisted in the mouse lens samples after γ-radiation exposure increased with decreasing dose-rate at 4 and 24 h. The C57BL/6 mice were exposed to 0.5, 1 and 2 Gy ƴ-radiation at 0.063 and 0.3 Gy/min and also 0.5 Gy at 0.014 Gy/min. This contrasts the data we obtained for peripheral blood lymphocytes collected from the same animal groups, which showed the expected reduction of residual 53BP1 foci with reducing dose-rate. These findings highlight the likely importance of dose-rate in low-dose cataract formation and, furthermore, represent the first evidence that LECs process radiation damage differently to blood lymphocytes.

## Introduction

Since the International Commission on Radiological Protection (ICRP) recommended a reduction in the eye lens occupational dose limit for ionising radiation (IR) exposure in 2012^1^ there has been a need for reliable mechanistic evidence to underpin the recommendations and subsequent changes in the IAEA and EU Basic Safety Standards which recently came into force^2^. Epidemiological studies supported the implementation of reduced dose limits^1,3,4^, however, the biological response of the lens and the mechanism(s) leading to IR-induced cataract remain unclear^5,6^. Historically, the focus of IR-induced cataract research has been on the effect of dose and threshold, to help decide whether cataract induction is most appropriately considered to be stochastic or deterministic (a ‘tissue effect’) in nature^6,7^. The effects of chronic, fractionated or protracted exposures remain unclear^8^, but recognition that the eye lens may be more radiosensitive than previously thought suggests there could be tissue-specific effects worthy of consideration, particularly in the contexts of medical and occupational exposures^3^.

Historically, dose rate has been considered in only a very few studies looking at cataract risk in IR exposed populations. A very recently published epidemiological study, by Little *et al*.^9^, has suggested an excess hazard ratio of 0.69 (0.27–1.16) per Gy of IR to the formation of cataract in radiologic technologists following cumulative occupational exposures to low doses (mostly < 100 mGy) accumulated over an average 5-year lagged eye lens absorbed dose^9^ (using self-reported cataract history as an endpoint). This excess hazard ratio was significantly different from the lowest dose exposure cases (<10 mGy cumulative exposure) used as a control group. Similar excess relative risk of 0.91 (0.67–1.20) has also been demonstrated to increase linearly during protracted exposures to ≥ 250 mGy from an analysis of the Mayak worker cohort^10,11^. Excess hazard ratios are difficult to compare between epidemiological studies due to a variety of factors including varying models and parameters. Nevertheless the authors found that the observed risks were statistically similar to those reported on the Japanese atomic bomb survivor cohort after reanalysis that estimated a lower excess hazard ratio of 0.32 (0.17–0.52) was reported^12^. However, the Little *et al*.^9^ study invovled a relatively large cohort of over 12,000 cases, alongside well characterised dosimetry – a key consideration for effective IR epidemiology^5,13^. Whilst there is little biological evidence for a dose-rate influence in the lens to date, it has been suggested as potentially having a large impact, particularly when doses are fractionated^14^. Thelatest epidemiological finding^9^ supports this suggestion, although the ICRP do not draw conclusions regarding the effect of dose-rate on the lens due to a current lack of sufficient evidence^1,15^.

The mechanism(s) of IR-induced cataract are still largely unknown, however, several possible mechanisms have been identified suggesting that a combination of processes likely play contributory roles^5^. The mouse is a highly appropriate *in-vivo* tool for such investigations on the eye lens for both early, short term biological effects^16–18^, as well as longer term studies of cataract formation^18–23^. LECs are organised as a single cell monolayer covering the anterior surface of the lens, where they are critical to lens function^24^. The epithelium can be spatially defined into two distinct regions, the central and peripheral regions; the latter consists of cells with a proliferative ability but not necessarily actively cycling^25,26^. The role of DNA damage and its potential impact on cataractogenesis in the lens is documented^16–18,22,27,28^. Notable examples include; cataractous lenses showing a high frequency of single strand breaks^28^, base and nucleotide excision repair genes having a suggested role in cataractogenesis^29^ and mutated *Xpd/Ercc2* genes (involved in DNA repair) result in sensitivity to IR^18^. Lens epithelial cells appear to employ both non-homologous end joining and homologous recombination to repair DNA damage, observed in the dose responses of both 53BP1 and Rad51 for both pathways respectively^17^. Haploinsufficiency for Atm and also Rad9 have been demonstrated to increase the radiosensitivity of the lens^22,30^.

IR-induced DNA double strand break (DSB) formation and repair can be quantified using a number of protein biomarkers, most commonly ƴ-H2AX, Rad51 or 53BP1^31–33^. IR-induced DNA damage assessed by ƴ H2AX and 53BP1 foci quantification in LECs of exposed lenses demonstrated a clear response at a range of doses^16,17^.

The dose rate at which IR is delivered is influential in the repair of DSBs. Low-LET IR is generally considered to be less effective when administered over a longer period, as opposed to an acute delivery, i.e. within minutes^34^. For decades it has been well understood that, certainly for low LET x- and ƴ-IR, a lowering of dose rate and extension of exposure time reduces the biological effect of a given dose particularly in cellular experiments^35^, and decreasing dose rate generally correlates to a decrease in the number of detectable un-repaired DSBs post-exposure^36^. Low dose rate is typically defined as being 5 mGy/hr or lower^37^. Results from studies using ƴ-H2AX demonstrate lower frequencies of residual DSB induced with lower dose rates in lymphocytes^38,39^. The study of Turner *et al*.^38^ compared two dose rates (1.03 Gy/min and 0.31 mGy/min), of x-irradiation and subsequent ƴ-H2AX foci yields in the peripheral blood lymphocytes of C57BL/6 mice. At 24 h post irradiation, the acute dose rate produced a significantly higher frequency of unrepaired damage in the lymphocytes from mice exposed to 1.1 and 2.2 Gy.

In this study, we explore the effect of varying dose-rates on residual 53BP1 foci in the lens of the eye following *in-vivo* exposure of female C57BL/6 mice. This strain is a reproducible mammalian model for investigating DNA damage and repair in the lens epithelium^16^. Adult mice 10 weeks of age were exposed^27^ and the effect of varying dose rates in LECs was measured using 53BP1 as a marker for IR-induced DNA DSB and repair. Peripheral blood lymphocytes were also collected from the same mice and analysed in parallel with the same marker to determine the comparative lens sensitivity to DNA damage resulting from three dose rates of IR exposure.

## Methods

A total of 78 female C57BL/6 (C57BL/6JOla/Hsd, Envigo RMS (UK) Ltd., Blackthorn, Bicester, Oxfordshire OX25 1TP) mice (142 lenses – 2 lenses omitted) were whole body exposed to IR at 10 weeks of age. Mice were exposed in groups of four for each dose, time and dose rate data point. Four mice (8 lenses) were used for each dose, time and dose-rate, with two mice for each point as controls (4 lenses), as per power calculations and previous observations on the reproducibility of this strain of mice^16^. Control mice were sham exposed, being transported to the exposure facility alongside the complimenting exposed mice. Six lenses were of insufficient quality to be analysed effectively. An additional 3 mice (6 lenses) were used for the 0.014 Gy/min exposures at 0.5 Gy. All procedures involving animals were performed in accordance with the Animals (Scientific Procedures) Act 1986 via approved project licencing granted by the UK Home Office, with additional approval from the local Animal Welfare and Ethical Review Body (AWERB) at Public Health England.

The exposures took place at the Medical Research Council Co-60 gamma irradiation facility (Harwell Campus, Didcot, Oxfordshire, UK)^40^, with a horizontal geometry. Doses of 0.5, 1 and 2 Gy were delivered at dose rates of 0.3, 0.063 and 0.014 Gy/min (Table 1). The irradiation system is calibrated and traceable to national standards. All doses were delivered to within 5% accuracy. Irradiations took place at room temperature, with whole body *in-vivo* exposure. A combination of 1, 2 or 4 cobalt-60 sources were used to achieve dose rates of 0.014, 0.063 and 0.3 Gy/min respectively.

**Table 1:** Table of exposure time for each scenario, matched to the date of exposure and source decay factor for that given time.

Dose (Gy)	300 mGy/min	63 mGy/min	14 mGy/min
0.5	1.5 min	7.1 min	39 min
1.0	3.1 min	14.4 min	N/A
2.0	6.3 min	29.2 min	N/A

**Figure 1:**
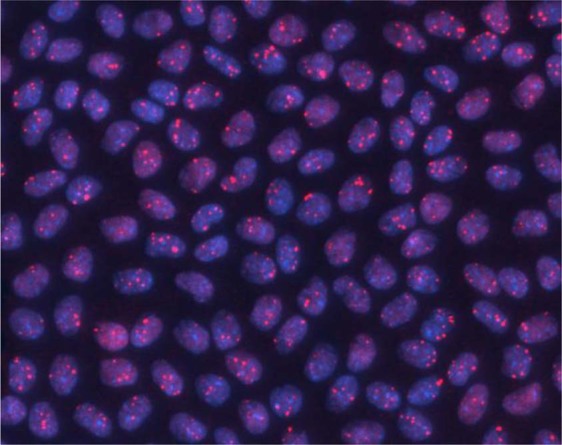
53BP1 foci in the nuclei of LEC located in the central region of the monolayer following 2 Gy irradiation (0.063 Gy/min) fixed and stained 4 h post-exposure.

Mice were sacrificed at 4 and 24 h post-exposure (from start of irradiation) and eyes fixed in formaldehyde. The techniques and methodologies for LEC preparation, isolation and immunofluorescence employed during this study have been described previously^16^ and were used in the current study, unless stated otherwise. Lens epithelia were isolated and immunofluorescence stained (Fig. 1) as described for 53BP1^16^. A minimum of 200 and 350 cells were scored for the central and peripheral regions, respectively per data point. From these initial data, power and sample size testing was carried out, based on a significance level of 0.05 and a power of 0.8 to ensure foci were scored in a large enough number of cells for comparative statistics to be applied^16^.

In addition to the lens epithelium, peripheral blood lymphocytes were also analysed from the same animals. Upon sacrifice, peripheral whole blood was extracted by cardiac puncture. This blood was placed on ice briefly to halt repair. The protocol used for lymphocyte extraction, fixation and immunofluorescent staining followed is described in previous publications^32,41^. Histopaque-1083 (Sigma-Aldrich, Germany) was used to isolate murine lymphocytes. A minimum of 200 nuclei were analysed for each of the peripheral lymphocyte samples using manual scoring.

Statistical analysis was performed using Minitab 17. General Linear Model Analysis of Variance (ANOVA) was applied to all data including dose, dose rate, epithelial region and time post exposure as factors. For the lens epithelial cell data, second and third order interactions for the variables dose, dose rate and time were explored to explain a relatively large lack of fit evident without this interaction. For dose and dose rate, plus the second order interaction between dose and time and the third order interaction between dose, dose rate and time, Tukey’s post hoc test for pairwise comparison between the different levels of these factors was then applied.

## Results

**Figure 2:**
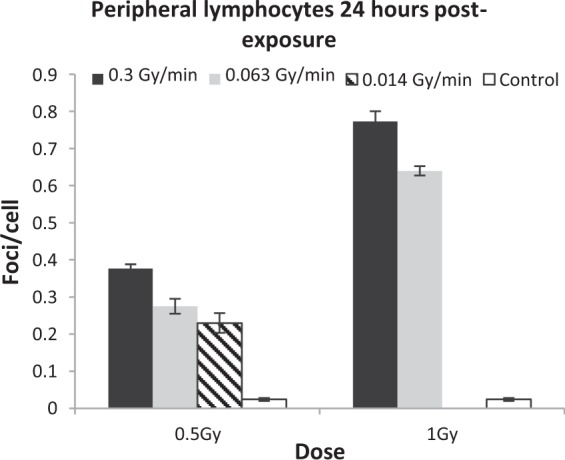
Mean 53BP1 foci/cell (including stand error bars) 24 h post-exposure to IR delivered with three different dose rates, measured in peripheral blood lymphocytes from *in-vivo* irradiated female C57BL/6 mice. As discussed in the main text, 0.014 Gy/min data is presented for 0.5 Gy only.

**Figure 3:**
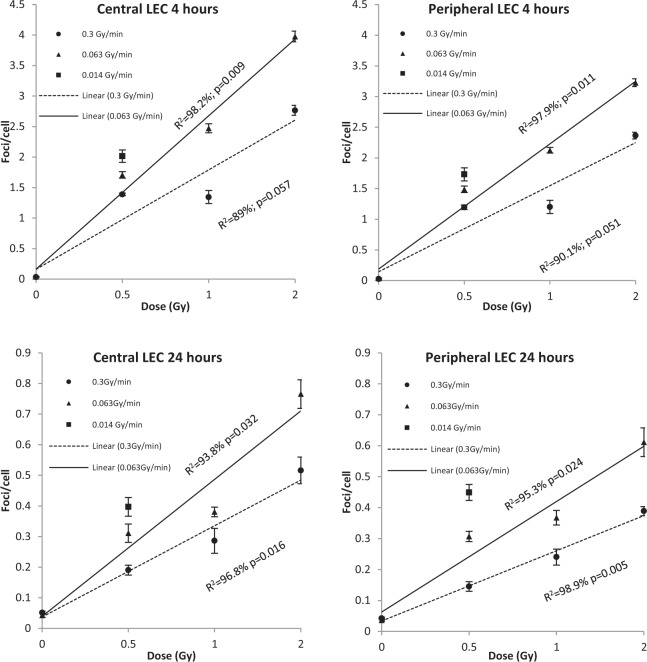
Mean 53BP1 foci/cell within central and peripheral region LEC (LEC) both 4 and 24 h post-irradiation to 0.5, 1 and 2 Gy (plus control) Co-60 ƴ-radiation at 0.3, 0.063 and 0.014 (0.5 Gy dose only as discussed) Gy/min dose rates. Note mean foci/cell axis is to a different scale at 24 h post-exposure compared to 4 h for ease of reading.

No significant effect of time, region, dose or dose rate in the sham-exposed control mice was observed. Analysis of the peripheral blood lymphocyte samples was performed firstly to compare with LECs, but secondly to confirm the dose rate effects seen in previous mouse studies^38^. Residual 53BP1 foci were scored at 24 h post-exposure for both 0.5 and 1 Gy doses at both 0.3 and 0.063 Gy/min dose rates and plotted with standard error (SE) bars (Fig. 2). All post-irradiation samples had more foci than unirradiated controls (p ≤ 0.001). At 0.5 Gy, the higher dose rate shows 0.376 (±0.011) mean foci/cell compared to 0.275 (±0.020) from the lower dose rate. At 1 Gy, the higher dose rate shows 0.773 (±0.027) foci/cell compared to 0.64 (±0.012) seen for the lower dose rate. Figure 2 demonstrates an even lower frequency of mean foci/cell (0.23 ± 0.026) at 0.5 Gy, delivered at 0.014 Gy/min, compared to both the 0.3 and 0.063 Gy/min dose rate exposures for 0.5 Gy. ANOVA reveals a significant influence of both dose and dose rate (p ≤ 0.001) on 53BP1 foci in lymphocytes.53BP1 foci were evaluated in the LECs of the central and peripheral regions, as in previous studies^16,17^. Mean foci frequencies were analysed at 4 and 24 h following exposure to doses of 0.5, 1 and 2 Gy irradiation at 0.3 and 0.063 Gy/min Co-60 ƴ-IR (Fig. 3). A reduction of mean foci/cell frequencies is seen at all doses between 4 to 24 h suggesting effective repair of DSBs. Doses delivered at 0.3 Gy/min yield fewer detectable foci than at 0.063 Gy/min in LECs. As mentioned, we included a lower dose rate of 0.014 Gy/min but were limited to only one dose, 0.5 Gy, due to mouse exposure time constraints. Figure 3 compares all three dose rates (0.014, 0.063 and 0.3 Gy/min) delivering a dose of 0.5 Gy *in-vivo*. The further reduction of dose rate (0.014 Gy/min) further increases detectable mean foci/cell frequencies in both regions at both 4 and 24 h post-exposure.

For statistical analysis, a variety of General Linear ANOVA Models were applied and finally a non-hierarchical model with a third order interaction for the variables dose, dose rate and time was used as this gave the best fit overall. Dose, dose rate and time were all found to significantly affect residual foci/cell yields (p ≤ 0.001), and the interaction between dose, dose rate and time was also significant (p < 0.001), indicating that DNA damage results must be considered in the context of dose, dose-rate and time post-exposure. Only the region of the epithelium ie peripheral versus central was not significant (p = 0.079), although significantly different residual foci yields were observed between regions previously at lower total doses and for X-ray rather than γ-ray exposures^16^. Tukey’s test then revealed that all doses were significantly different from each other, apart from 0.5 and 1 Gy (p = 0.339). For all doses, the intermediate dose rate of 0.063 Gy/min shows a significantly higher frequency of mean foci/cell compared to the higher dose rate of 0.3 Gy/min (p = 0.001) and at 0.5 Gy the residual foci yields at the lower dose rate of 0.014 Gy/min was significantly different from those at 0.3 Gy/min (p = 0.014). There was no significant difference between the two lower dose rates (p = 0.696) at 0.5 Gy 24 h after exposure.

## Discussion

The observed inverse dose-rate effect in LEC is evident in both 4 and 24-hour post-exposure data. The limited biological evidence of an inverse dose rate effect in the lens has recently been summarised by Hamada *et al*.^14^, suggesting there might be a dose-rate effect at low-LET exposures. Moreover this inverse dose-rate effect would only observed with high-LET neutron radiation^42^. It should also be noted that standard errors associated with mean foci/cell are small, a previously observed characteristic of both the radiosensitive Balb/c and the less sensitive C57BL/6 strains^16^. Further, post hoc testing demonstrates a clear trend of higher foci numbers with higher doses, shorter times and, to some extent, lower dose rates. Dose rate reduction, and increased duration of exposure generally reduce the biological effects of ionising radiation^35^ as observed in the lymphocytes of both this study and in Turner *et al*.^38^, where less DNA damage foci were detected.

The somewhat unexpected finding of this study may be explained when considering the interaction effects. This additional feature of the observed data is the significant interaction effect, i.e., the statistically significant interaction between time (post-exposure), dose and dose-rate, as well as between dose and time, both with p-values of < 0.001. Such an interaction is not something commonly considered during such analyses, but the results here are clear. This analysis was justified due to the large error observed within the sum of squares from ANOVA testing, when the interaction effect was not included. Dose, dose rate and time are biologically known to have interactions and indeed it appears from out data that 53BP1 foci frequencies are influenced by all three.

The dose rates used in this study are not low in comparison to the generally accepted definition of <5 mGy/h^43^, below which single track events will predominate. The choice of dose rates in this study was driven chiefly by the availability of suitable exposure facilities and the dose and dose rate ranges within which the source could be reliably calibrated. However, the data presented here support the hypothesis^8,9^ that there is a dose rate effect in the lens and not in the lymphocytes. Reducing dose rate from 0.3 to 0.063 Gy/min was found to significantly increase the number of detectable residual DSBs within LEC DNA, which raises the question of the effects of protracted low dose-rate exposures on the lens. More investigations are required to establish the influence of both IR dose-rate and also energy on the DNA damage response of LECs.

Whilst this study considers one response to IR exposure, i.e. DNA damage assessed by 53BP1 foci, recent findings suggest it has a role in IR-induced cataractogenesis^16–18^. Based on the dose responses seen for both Rad51 and 53BP1 foci in a previous study, both homologous recombination and non-homologous end joining repair mechanisms, respectively, appear to be active in LECs^17^. it has been suggested that a combination of IR-induced effects is responsible for cataract induction^5,44^ and our data support this view. Un-repaired DNA damage in LEC could lead to altered cell proliferation^17^, which could in turn have a detrimental effect on cell differentiation. Our data also suggest that gamma-ray IR induced damage may accumulate as evidenced by the persistence of foci 24 h after exposure across the lens epithelium (Fig. 3). The presence of progenitor cells in the lens epithelium^45–47^ would potentially pose a particularly sensitive target for IR-induced DNA damage. Another possible mechanistic explanation for the results of this study could relate to the roles of lens epithelium-derived growth factor (LEDGF) and eyes absent (EYA) proteins. LEDGF is expressed in greater concentration across the central region of the lens epithelium^48^, where it has been shown to protect the cells against oxidative damage^49^ and DNA damage induced via peroxide and UVB^50,51^. The expression of LEDGF is lost towards the equator of the lens, where the peripheral region LECs are located^48^ resulting in slower repair. This is important to our study as LEDGF has DNA binding properties^52^ and is involved in the DNA DSB repair response pathway following γ-radiation exposure^53^. Recent proteomic and RNA sequencing data have revealed, higher levels of DNA repair proteins 53BP1, MRE11 and Rad50 in the murine lens epithelium compared to fibre cells^54^, but the fact that the peripheral epithelium is more sensitive to IR^17^ suggests that LEDGF might play a key role in this differential sensitivity between the central and peripheral regions of LECs. EYA, a phosphatase required along with Pax6 and Six3 transcription factors that are essential for eye development^55^, is also needed for DNA repair as it dephosphorylates H2AX^56^. Both LEDGF^49,57^ and EYA^58^ are also required for LEC differentiation into LFCs. Our data indicate that DNA repair is dependent upon dose and dose rate. We interpret this to indicate that peripheral LEC are balancing the processes of differentiation and DNA DSB repair. At the low dose rates, the 53BP1 foci persist whilst differentiation proceeds, but at higher dose rates, DNA repair is favoured over differentiation. The decreased expression of LEDGF in the peripheral region LEC could help explain the slower repair of DNA damage seen in our study at the lower dose rate (Fig. 3). The 53BP1 foci indicates that DNA repair is progressing, as we also observe a decrease in foci/cell with time for both peripheral and central regions of the lens epithelium.

Inverse dose-rate effects generally occur in instances whereby a continuous low dose rate exposure to IR allows cells to cycle, then hitting a G2-phase block, resulting in cell death^35^. However, cell death does occur in LECs but this is very low, and there are no reported instances of increased LEC death post-IR exposure^5^. Lifetime loss of LEC via cell death in humans over many decades may have some effect on LEC differentiation and lens transparency^59^. One proposed mechanism of the inverse dose-rate effect is a failure of LECs to arrest damaged cells and halt their progression into G2 cell cycle phase. This would make the periphery a particular target of the inverse dose-rate effect^14^ as it is here that proliferating cells are concentrated^60^. A consequence of delaying DSB repair is that secondary effects accrue and affect lens cell differentiation itself^6,14^. This is broadly summarised in the ‘cataractogenic load’ concept, where genetic, lifestyle and environmental factors are recognised as con-inducers of age-related cataract^44^. IR-induced damage therefore compounds the lens aging process by further increasing this ‘load’ to accelerate aging and the appearance of age-related cataract. Our report here of an inverse dose rate effect fuels further debate on IR effects upon the lens and strengthens arguments for regular monitoring in occupational contexts^3,61,62^.

## Conclusion

This study represents the first *in-vivo* experimental investigation of dose-rate effects in the epithelial cells of the mouse eye lens and presents the first evidence to suggest that LEC and lymphocytes respond differently to dose-rate variation in terms of DNA damage repair. Levels of residual DNA damage foci observed in LECs increase with reduced dose-rate unlike in peripheral lymphocytes from the same animals. This supports the hypothesis that the lens has an unusual DNA damage response to IR compared to other tissues. Our data indicate an inverse dose rate effect in the LEC for residual 53BP1 foci as a marker for DSBs, and it is hypothesised that this residual damage contributes to cataractogenesis. This study evidences the importance of determining the cell-specific effects of low dose and low dose-rate IR exposures on DNA repair in target tissues and show that the response can be cell and tissue specific due to for instance, competing processes such as cell proliferation and differentiation in a dose-rate dependant manner as observed in cell-based studies^63–65^.

### Ethical approval and informed consent

All experimental protocols were approved by the UK Home Office and approved under project licencing, as well as local Animal Welfare and Ethical Review Body. Methods were carried out in accordance with the relevant guideline and regulations stated above.

## Data Availability

The data generated during the current study are available from the corresponding author on reasonable request.
